# Theta and Alpha Oscillations during the Retention Period of Working Memory by rTMS Stimulating the Parietal Lobe

**DOI:** 10.3389/fnbeh.2017.00170

**Published:** 2017-09-14

**Authors:** Song Li, Jing-Na Jin, Xin Wang, Hong-Zhi Qi, Zhi-Peng Liu, Tao Yin

**Affiliations:** ^1^Institute of Biomedical Engineering, Chinese Academy of Medical Sciences & Peking Union Medical College Tianjin, China; ^2^Neuroscience Center, Chinese Academy of Medical Sciences Beijing, China; ^3^Laboratory of Neural Engineering and Rehabilitation, Institute of Precision Instrument and Opto-Electronics Engineering, Tianjin University Tianjin, China

**Keywords:** rTMS, EEG frequency, retention period of working memory, parietal lobe, phase synchronization

## Abstract

Studies on repetitive transcranial magnetic stimulation (rTMS) have shown that stimulating the parietal lobe, which plays a role in memory storage, can enhance performance during the “retention” process of working memory (WM). However, the mechanism of rTMS effect during this phase is still unclear. In this study, we stimulated the superior parietal lobe (SPL) using 5-Hz rTMS in 26 participants and recorded electroencephalography (EEG) while they performed a delayed-recognition WM task. The analyses included the comparisons of event-related spectral perturbation (ERSP) value variations in theta (4–7 Hz) and alpha (8–14 Hz) band frequencies between conditions (rTMS vs. sham), as well as the correlations between different brain areas. Following rTMS, the ERSP values of theta-band oscillations were significantly increased in the parietal and occipital-parietal brain areas (*P* < 0.05*), whereas the ERSP values of alpha-band oscillations were significantly decreased in the parietal area (*P* < 0.05*). The ERSP value variations of theta-band oscillations between the two conditions in the left parietal and left prefrontal areas were positively correlated with the response time (RT) variations (by using rTMS, the more subject RT decreased, the more ERSP value of theta oscillation increased). The ERSP value variations of alpha-band oscillations in the left parietal and bilateral prefrontal areas were negatively correlated with RT variations (by using rTMS, the more RT of the subject decreased, the more ERSP value of alpha oscillation decreased). Inter-sites phase synchronization of theta-band EEG between the left parietal and left prefrontal areas, as well as alpha-band EEG between the left parietal and bilateral prefrontal areas were enhanced by rTMS. These results indicated that activities of both parietal and prefrontal areas were required for information storage, and these activities were related to the behavioral responses. Moreover, the connectivity between these two regions was intensified following rTMS. Thus, rTMS may affect the frontal area indirectly via the frontal parietal pathway.

## Introduction

The working memory (WM) process could be divided into three phases; encoding, retention and information recall (Baddeley et al., 2001). Many studies have investigated the maintenance and recall of stored information in the “retention” process. The brain activity during retention period was observed when subjects performed a delayed-recognition task, and had shown that multiple cortical areas, such as the prefrontal and parietal brain regions were activated. Some studies have demonstrated contributions from the prefrontal brain region to maintenance of stored information during the “retention” process (Gevins et al., 1997; Jensen and Tesche, 2002; Meltzer et al., 2008; Hsieh and Ranganath, 2014), while other studies implicate activated parietal regions during the process (Raghavachari et al., 2006; Jensen and Mazaheri, 2010; Hsieh et al., 2011).

Electroencephalography (EEG) oscillation has been used as an important index for evaluating brain activity. Specially, EEG theta (4–7 Hz) and alpha (8–14 Hz) band oscillations were extensively used to reflect brain activities during WM. Many studies observed variations in theta oscillation during WM (Raghavachari et al., 2001; Scheeringa et al., 2009; Hsieh and Ranganath, 2014; Kardos et al., 2014). For the “retention” process, some studies have shown association of increased theta band power in EEG recordings with increased WM load (Tesche and Karhu, 2000; Jensen and Tesche, 2002; Meltzer et al., 2008; Hsieh et al., 2011). Meanwhile, EEG alpha band oscillation is regarded as the dominant oscillatory activity of the human brain, and has been associated with basic cognitive functions such as attention and memory (Klimesch et al., 2007). Some studies demonstrated EEG alpha band power changes with increased memory load (Krause et al., 2000; Klimesch et al., 2003). Moreover, during the “retention” process, another function that has been attributed to alpha activity is a mechanism of functional inhibition at neuronal level for gating of information in the task-irrelevant brain areas (Michels et al., 2008; Jensen and Mazaheri, 2010). Alpha activity thus plays an important role in attention by supporting processes within the attentional focus, while blocking those outside this focus.

Recently, high-frequency (>1 Hz) repetitive transcranial magnetic stimulation (rTMS) has increasingly been used in the neurocognitive field as a non-invasive method to study brain activities (Luber and Lisanby, 2014). Specific cortex can be activated by stimulating the appropriate regions of interest (ROI) in the brain during the retention period of WM tasks in order to explore the role of various cortices implicated in storage (Postle et al., 2006; Luber et al., 2007; Hamidi et al., 2008). These studies have revealed that rTMS specifically to the parietal cortex alters WM performance, suggesting a preferential role for the parietal cortex in memory storage. Luber et al. (2007) applied rTMS to either left dorsolateral prefrontal or midline parietal brain regions, and found that only stimulation to the parietal brain region resulted in a significant decrease in response time (RT) of WM without a corresponding decrease in accuracy. Moreover, there was a shorter response period associated with right stimulation after activating the parietal cortex bilaterally (Yamanaka et al., 2009), suggesting that the parietal cortex was involved in information storage in WM.

According to previous studies, the frequency of stimulation also plays an important role in the effect of rTMS in memory storage. Some studies stimulated the parietal region during the retention process with a stimulation frequency in the alpha band, and found that rTMS could increase the accuracy rate (AR; Klimesch et al., 2003; Hamidi et al., 2009). The investigators conceived that rTMS enhanced the capacity of inhibition of interfered information in the task-irrelevant brain areas. Moreover, many studies considered the theta-band rTMS, whereby the stimulation frequency lies in the theta band. Luber et al. (2007) used 1-Hz, 5-Hz and 20-Hz rTMSs to stimulate the parietal region, and found that the RT could be reduced by only the 5-Hz rTMS. Yamanaka et al. (2009) again showed that stimulating the parietal area via 5-Hz rTMS could reduce the RT of WM. They concluded that the behavior enhancement by the 5-Hz rTMS was due to a temporary increase in amplitude of the excitatory postsynaptic potentials, and possibly the rTMS was affected by the resonance between the EEG oscillation (e.g., theta band oscillation). However, there was no definite evidence about neurophysiological effects of 5-Hz rTMS during the “retention” process of WM, or its underlying mechanisms may still remain unclear.

In studies of brain activity during the “retention” period of WM, there are few reports on the combination of rTMS evoked features, EEG rhythms, and behavioral findings. Therefore, the aim of this study was to investigate the effects of 5-Hz rTMS in the parietal brain region during the “retention” period of WM. A classical “Sternberg paradigm” verbal “delayed-recognition” task was used in this study. The WM experimental procedure was similar to that reported by Luber et al. (2007). In order to investigate EEG activities in the parietal brain region affected by rTMS, we focused on: (1) The contribution of theta and alpha band oscillations to the retention of WM performance, (2) The correlations between the oscillatory theta or alpha response, rTMS induction and performances (e.g., AR and RT changes) of participants, and (3) The inter regional synchronization of oscillations among the region of interest (e.g., parietal area, prefrontal area) after rTMS. For this study, we hypothesized that the 5-Hz rTMS could induce changes in EEG oscillations (e.g., theta and alpha bands) in the brain areas, and these changes will be related to behavioral performances. Moreover, inter-sites EEG phase synchronizations between the prefrontal and parietal cortices would be enhanced by rTMS.

## Materials and Methods

### Subjects

This study involved 26 participants (18 men and 8 women; mean age: 28 years, SD = 2.1). Participants were graduate students without any mental or neurological diseases or related family history of disease. This study was carried out in accordance with the recommendations of the local ethics committee with written informed consent from all subjects. All subjects gave written informed consent in accordance with the Declaration of Helsinki. The protocol was approved by the local ethics committee.

### Procedure

**Figure 1:**
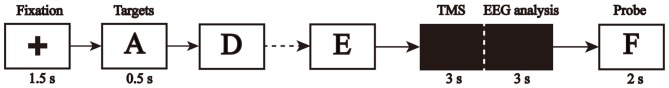
Flow diagram of the single-pass working memory (WM) test of letters.

The task was designed using E-prime software (Eprime2.0, Psychology Software Tools, Inc., Sharpsburg, PA, USA). We used the classical Sternberg paradigm to design the letter WM experiment. The participants were instructed to sit in front of a computer screen, and keep their gaze at the center of the screen. The letter memory experiment protocol is shown in Figure 1. First, a symbol, “+”, appeared on the screen for 1500 ms to alert the subjects, followed by five target letters, which appeared one at a time for 500 ms each. Participants were asked to memorize all the target letters (memory coding stage). Next, a black screen appeared for 6000 ms, while participants tried to recall the letters (retention period). Test letters were presented on a black screen for 2000 ms each, and participants were asked to judge whether the letter was the same as one of the memorized target letters; they pressed “1” for “yes” and “2” for “no” (memory matching stage). The rTMS was applied at the beginning of the memory maintenance stage for 3000 ms. EEG data obtained after applications of the rTMS were used for subsequent EEG analysis.

There were six groups in the experiment and 20 trials in each group. The interval time between groups was 2 min, and that between trials was 10 s. Half of the groups received rTMS, whereas the other half received sham rTMS. All the groups were randomized so that participants did not know which treatment they received.

### rTMS Procedure

For the rTMS session, participants were seated comfortably and their heads were localized in space via a three-dimensional navigation system (Brainsight, Magstim, Carmarthenshire, UK). Prior to the start of the behavioral task, resting motor threshold (RMT) was determined for each participant using electromyography (Matrix, Micromed, Mogliano Veneto, Italy). The target for RMT was the left primary motor cortex, which controls the right first dorsal interosseous muscle. RMT was determined as the minimum stimulus intensity capable of eliciting muscle activity of more than 50 μV in at least 5 of 10 consecutive TMS.

We used the RMT to calibrate the stimulation intensity for each subject, starting at 110% of RMT and accounting for scalp-to-cortex distance for each targeted brain area (Stockes et al., 2006). A 70-mm figure-of-eight coil (Double 70 mm Coil, Magstim) was used during the first 3000 ms of the maintenance stage (total 15 plus) for the 5-Hz rTMS, which is similar to theta band frequency. To avoid sound interference produced by rTMS, participants were required to wear earplugs. The location of the coil was tangential to the scalp, and the hand shank was vertical to the gyrus of the target cortex. In the “sham” condition, a similar rTMS coil was applied, which was located in the same position.

Prior to determining the target location from the cortex, whole-brain images were acquired with a 3T scanner (Sigma VH/I, GE Medical Systems, Milwaukee, WI, USA). High-resolution T1-weighted images (256 axial slices, 0.5 mm × 0.5 mm × 0.8 mm) were obtained for all participants. This scan was used to reconstruct a 3-dimensional image of each participant’s head, which was then used to target rTMS to the superior parietal lobule (SPL). The SPL is located between the posterior central gyrus and the intraparietal sulcus (middle area between the P3 and P1 electrodes near Brodmann area 7).

### EEG Recording

A 32-guide EEG acquisition system (Synamps2, CompumedicsNeuroscan, Charlotte, NC, USA) with a titanium alloy electrode cap (Maglink, CompumedicsNeuroscan) to shield from rTMS interference was used to record EEG. We used the International 10–20 system to apply electrodes (FP1, FP2, F7, F3, Fz, F4, F8, T7, C3, Cz, C4, T8, TP7, CP5, CP3, CP1, CPz, CP2, CP4, CP6, TP8, P7, P5, P3, P1, Pz, P2, P4, P6, P8, O1 and O2). The ground electrode was located in front of the forehead, and reference electrode was placed in the middle of the “Cz” and “CPz” electrodes. Vertical and horizontal eye movement electrodes were also applied. All electrode impedance values were <5 kΩ. The sampling rate was 1000 Hz.

### Data Analysis

#### Pre-Processing

We used EEGlab v12.0 (EEGlab, San Diego, CA, USA; Delorme and Makeig, 2004) to pre-process the EEG data. The reference electric potential was changed into the average potential of all the electrodes. The EEG signal was down-sampled to 500 Hz, and the pass-band was adjusted between 0.15 Hz and 100 Hz using the finite impulse response (FIR) digital filter. TMS-evoked electrical artifacts were reported as the main source of interference in previous rTMS studies (Veniero et al., 2009; Rogasch et al., 2014). In this study, we analyzed the terminal 3-s EEG signal after rTMS application during the “retention period” of WM. Moreover, the independent component analysis (ICA) method was used to reject artifact components (Hamidi et al., 2010). This process included two stages. In the first stage, ICA was performed on continuous EEG data, and an average of 5.5 ± 2.1 rTMS artifact-related components were identified and removed. After that, the continuous EEG data was split into many epochs. Each epochs included a complete memory experiment procedure (last 12 s) and the interval time between the two trials (last 10 s) which was used for the inter individual normalization. In the next stage, a second ICA was performed on data from each epoch, and an average of 2.1 ± 1.4 rTMS artifact-related components were identified and removed. Furthermore, an average of 2.0 ± 0.5 artifact-related ICA components due to muscle movements and blinking were also identified and removed. After the EEG pre-processing, each subject had an average of 55.38 ± 0.94 epochs for statistical analysis.

#### Spectral Analysis

Some researchers have demonstrated the event-related spectral perturbation (ERSP) value variation in EEG oscillations during the “retention” periods, such as the increased ERSP value of theta oscillations (Sauseng et al., 2004, 2010; Raghavachari et al., 2006; Sauseng and Klimesch, 2008) and the decreased ERSP value of alpha oscillations (Klimesch et al., 2003). Therefore, we used the ERSP (Makeig, 1993) to analyze the ERSP value variation of the brain oscillations.

The ERSP measures average dynamic changes in amplitude of the broad band EEG frequency spectrum as a function of time relative to an experimental event. These spectral changes typically involve more than one frequency or frequency band (i.e., theta band or alpha band). To compute an ERSP, baseline spectra are calculated from the EEG immediately preceding each event. The epoch is divided into brief, overlapping data windows, and a moving average of the amplitude spectra of these is created. The ERSP was calculated by dividing the respective mean baseline spectra from each of the spectral transforms of individual response epoch. The ERSPs for many trials are then averaged to produce an average ERSP, plotted below as relative spectral log amplitude on a time-by-frequency plane.

ERSPs were computed using a moving Hanning-window wavelet with three cycles for the lowest frequency (4 Hz) and increasing linearly to 30 cycles for the highest frequency (80 Hz) analyzed. In this study, the “retention” period lasted 6 s. The ERSP analyses were performed over a time period from 1000 ms prior to the onset of the “retention” period to the end of the epoch. The baseline period was 1000 ms prior to the onset of the “retention” period. However, during the “retention” period only the terminal 3-s EEG signal (after the rTMS application) was used for the analysis. Mean retention period ERSP was calculated separately for each subject and each electrode.

Responses were normalized for each participant by subtracting the calculated mean ERSP from that of a 2-s period within interval between the two trials for that participant. To extract the ERSP data during the interval, we also performed an ERSP analysis over a time period from 1000 ms prior to the onset of the “retention” period to the beginning of the next trail (before the next symbol “+” appeared). The baseline period was also 1000 ms prior to the onset of the “retention” period. We select and average the ERSP data of 2 s in the middle of the interval (during 10 s between the two trails) for the interval-period ERSP data. The normalized ERSP data were the ERSP data during the “retention” period subtracted the interval-period ERSP data.

#### Spectral Correlations with Behavior

In rTMS-EEG research, it was found that there was a negative correlation between RT and alpha oscillation ERSP values after application of 10-Hz rTMS to the left parietal region during the “retention” process (Hamidi et al., 2009). Pearson’s correlation coefficient (Eggers et al., 2003) was used to estimate the correlation between the spectra from the special frequencies of the EEG signals and the behavior performances. For all brain areas, the differences between the two conditions (rTMS vs. sham) for theta band or alpha band were calculated for each subject. These different values were correlated with two behavior parameters (RT or AR) to determine which brain areas were significantly correlated with the WM tasks.

#### Phase Synchronization

EEG phase differences are often used to investigate “inter-sites phase synchronization”, which is an estimation of the connectivity between two EEG electrode sites (van Mierlo et al., 2014; Kang et al., 2015; Tokariev et al., 2016). Phase locking value (PLV; Lachaux et al., 1999) is a possible means to represent the synchronization phenomena in EEG signals (Lachaux et al., 1999). To obtain amplitude envelopes, absolute values were taken from Hilbert-transformed signals. PLV, which measures the variability of phase difference in a time interval, is calculated as follows:

(1) $$PLV_{t}=\frac{1}{N}\sum_{n=1}^{N}\left|\langle \exp(i(\varphi_{chan1}^{n}-\;\varphi_{chan2}^{n}))\rangle \right|$$

where *N* is the number of time points in a time window *t*, *φ^n^* denotes phase from a given channel at a time-point *n*, and *i* is the imaginary unit.

The range of PLV values vary between 0 and 1. A PLV value of 1 indicates perfect coupling of electrode pairs, whereas a PLV value of 0 indicates lack of electrode pair coupling. In this study, PLV was calculated using a sliding window of 1500 points (3000 ms) length. To obtain the estimates of PLV time courses for each electrode (e.g., chan1), PLV time courses between chan1 and another electrode (e.g., chan2) were averaged. The PLV was computed between the two electrodes sited near the five brain areas in both theta and alpha oscillations. The five brain areas included the frontal area (FP1, FP2, F7, F3, Fz, F4 and F8) the central cerebral area (T7, C3, Cz, C4 and T8), the central-parietal area (TP7, CP5, CP3, CP1, CPz, CP2, CP4, CP6 and TP8), the parietal area (P7, P5, P3, P1, Pz, P2, P4, P6 and P8), and the occipital area (O1and O2). A statistical method was used to assess the significant differences between rTMS and sham.

### Statistical Analysis

For statistical analysis of behavioral data, the paired *t*-test was adopted. The study detected significant differences for the “AR” and the “RT” of subjects between the two conditions, and evaluated the effects of rTMS on parietal brain regions. Bonferroni correction was used for statistical result adjustments.

Permutation test was conducted to test hypothesis as stated in the “Introduction” Section. A permutation test is usually used to assess statistical significance. As a test based on computer simulation, permutation test is especially suitable for small sample data sets, such as nonparametric tests. Moreover, permutation test provides an efficient approach to testing when the data do not conform to the distributional assumptions of the statistical method one wants to use (e.g., normality). The theory for permutation test has evolved from the works of Fisher (1935) and Pitman (1938). Pesarin (1992) developed the method, and the principle is as follows.

First, assuming two samples were assigned as X1= (*x*_11_, *x*_12_…*x*_1*n*1_) and X2 = (*x*_21_, *x*_22_…*x*_2*n*2_). Where *n*_1_ and *n*_2_ are the numbers of samples (X1 and X2). Second, the statistics of the original observations *T*_0_ were calculated. Third, the order of the sample data is replaced by a large number of random permutations to calculate the new statistics $T_{i},\;\left(i=1,2,\ldots ,C=\left(\begin{array}{c} n_{1}+n2\\ n_{1} \end{array}\right)\right)$. The *P* value of the statistical test is calculated by formula:

(2) $$p=\text{Prob}(T\geq T_{0})=\frac{1}{C}\sum_{i=1}^{C}I(T_{i}^{perm}>T_{0})$$

In this study, the characteristics of the results from the spectral and inter-sites phase synchronization analysis were consistent with the requirements of permutation test (small sample size (26 participants), and not satisfying normal distribution). Therefore, the permutation testing was used to evaluate significant differences of EEG analysis data between the rTMS and sham conditions. The statistical results were Bonferroni corrected.

For the spectral analysis, all of the electrodes (32 electrodes) were involved in the statistical analysis. The ERSP data were averaged both in time (retention period) and frequency (theta band and alpha band) domains in each electrode. The permutation testing was used to evaluated the significant difference between the two conditions (ERSP-rTMS and ERSP-sham).

For the phase synchronization analysis, there are about 496 electrode pairs (due to the 32 electrodes) were involved in the statistical analysis. The PLV data calculated for each electrodes pair. The permutation testing also was used to evaluate the significant difference between the two conditions (PLV-rTMS and PLV-sham).

## Results

### Behavioral Results

The behavioral results from the WM tasks included AR and RT values. The Pauta criterion was imposed to reject abnormal trials as follows: if the difference between the RT value from one trial and the averaged RT value for all trials was larger than three times the standard deviation (SD) of the averaged RT, then the trial was rejected. Using this criterion, 2 ± 0.8 trials were rejected in one participant.

**Figure 2:**
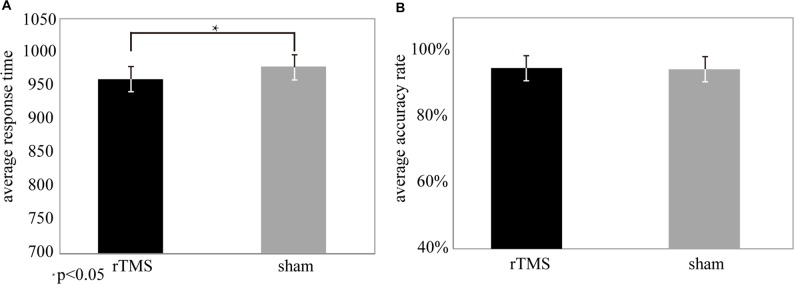
Behavioral data from the repetitive transcranial magnetic stimulation (rTMS) and sham conditions. **(A)** Response time (RT) was significantly different (*t*_(25)_ = 3.5, *P* < 0.05*) for the rTMS (mean (M) = 908.69, standard deviation (SD) = 12.56) and sham (*M* = 947.36, SD = 15.25) conditions. **(B)** The accuracy rate (AR) was not significantly different (*t*_(25)_ = 2.1, *P* > 0.05) between the rTMS (*M* = 94.5%, SD = 0.04) and sham (*M* = 92.8%, SD = 0.05) conditions.

Paired *t* test showed that AR values were not significantly different between the rTMS and sham groups (*t*_(25)_ = 2.1, *P* > 0.05, see Figure 2). In contrast, RT values were significantly shortened in the rTMS group compared to the sham group (*t*_(25)_ = 3.5, *P* < 0.05*, see Figure 2), which indicated that rTMS had effectively improved the task performances.

### Effect of rTMS

**Figure 3:**
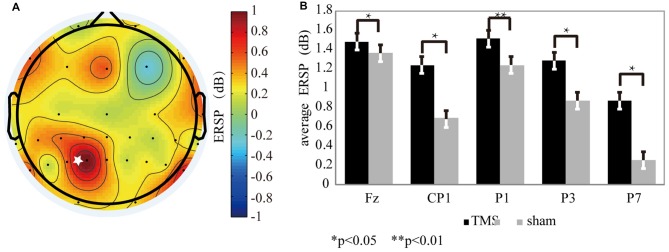
The theta oscillation variation for the rTMS and sham conditions. **(A)** The theta oscillation mapping was obtained by subtracting the sham condition from the rTMS. From the map, regions with increased energy include the parietal and prefrontal areas. The “*” symbol represents the stimulation location. **(B)** The electrodes showing significantly different readings included Fz (*P* < 0.03*), P7 (*P* < 0.02*), P3 (*P* < 0.05*), P1 (*P* < 0.01**), and CP1 (*P* < 0.04*).

**Figure 4:**
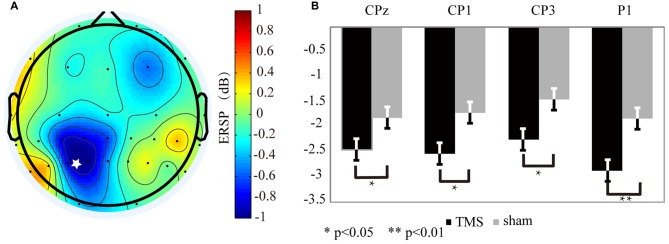
The alpha oscillation variation for the rTMS and sham conditions. **(A)** The alpha oscillation mapping was obtained by subtracting the sham condition from the rTMS. From the map, only the left parietal area exhibited decreased power. In contrast, power increased in the contralateral region. The “*” symbol represents the stimulation location. **(B)** The electrodes showing significantly different readings included CP1 (*P* < 0.03*), P1 (*P* < 0.01**), CP3 (*P* < 0.04*), and CPz (*P* < 0.05*).

For the theta oscillation, ERSP results showed that there was a significant difference between the rTMS and sham groups. The ERSP values of theta oscillations (Figure 3A) were increased markedly by rTMS treatment in the left SPL area, which included P7 (*P* < 0.03*), P3 (*P* < 0.05*), P1 (*P* < 0.01**), and CP1 (*P* < 0.04*; Figure 3B). Similar result was found near the prefrontal area, except that only the ERSP values from the Fz (*P* < 0.03*) electrode was significantly increased with rTMS (Figure 4B).

In contrast to the theta band results, the EEG mapping (Figure 4A) for the alpha band showed that, the ERSP values in the left SPL region, which included CP3 (*P* < 0.04*), CP1 (*P* < 0.03*), CPz (*P* < 0.05*) and P1 (*P* < 0.01**) markedly declined with rTMS effect (Figure 4B).

### rTMS Correlated with Behavior

**Figure 5:**
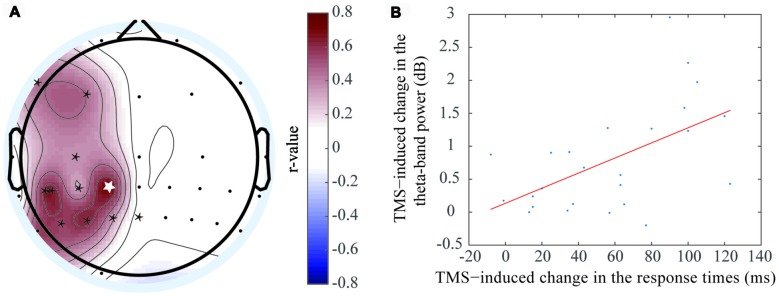
Positive correlation was exhibited between the event-related spectral perturbation (ERSP) value difference of the theta oscillation and response time (RT) difference. **(A)** The electrodes with significant positive correlations included F7 (*r* = 0.425, *P* < 0.05*), F3 (*r* = 0.451, *P* < 0.05*), C3 (*r* = 0.356, *P* < 0.05*), CP3 (*r* = 0.621, *P* < 0.05*), CP1 (*r* = 0.715, *P* < 0.01**), CP5 (*r* = 0.657, *P* < 0.01**), P5 (*r* = 0.546, *P* < 0.05*), P3 (*r* = 0.553, *P* < 0.05*), P1(*r* = 0.625, *P* < 0.05*), and Pz (*r* = 0.356, *P* < 0.05*). **(B)** The correlation at the CP1 electrode, which was shown in figure **(A)** with the white asterisk.

**Figure 6:**
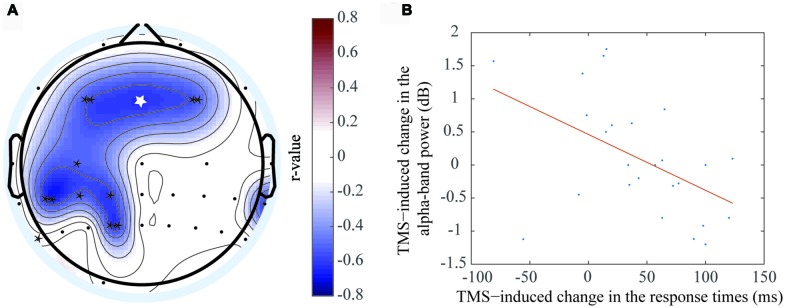
Negative correlation was exhibited between the ERSP value difference of the theta oscillation and RT difference. **(A)** The electrodes with significant negative correlations included F3 (*r* = −0.559, *P* < 0.01**), Fz (*r* = −0.726, *P* < 0.05*), F4 (*r* = −0.535, *P* < 0.01**), C3 (*r* = −0.527, *P* < 0.05*), CP1 (*r* = −0.607, *P* < 0.05*), CP3 (*r* = −0.52, *P* < 0.05*), CP5 (*r* = −0.661, *P* < 0.01**), P7 (*r* = −0.601, *P* < 0.05*), P1 (*r* = −0.638, *P* < 0.01**). **(B)** The correlation at the Fz electrode, which was shown in figure **(A)** with the white asterisk.

Due to individual differences in brain capacity, performances on WM tasks differed across participants. The RT differences were significantly correlated with the EEG ERSP values differences between the rTMS and sham groups for the theta (Figure 5A) and alpha (Figure 6A) oscillations.

A positive correlation was observed at the theta frequency band, which indicated the theta oscillation ERSP values increased when the RT differences increased between the rTMS and sham groups. This phenomenon was localized mainly to the left brain regions, including the left prefrontal area, left central area, left central-parietal area and left parietal area. The electrodes that had the significant positive correlations by rTMS treatment included F7 (*r* = 0.425, *P* < 0.05*), F3 (*r* = 0.451, *P* < 0.05*), C3 (*r* = 0.356, *P* < 0.05*), CP3 (*r* = 0.621, *P* < 0.05*), CP1 (*r* = 0.715, *P* < 0.01**), CP5 (*r* = 0.657, *P* < 0.01**), P5 (*r* = 0.546, *P* < 0.05*), P3 (*r* = 0.553, *P* < 0.05*), P1 (*r* = 0.625, *P* < 0.05*), and Pz (*r* = 0.356, *P* < 0.05*). The most marked difference was observed at CP1 (Figure 5B), which was located at the left parietal area. Previous studies had shown that object memory was associated with the left-brain cortex. Thus, this result suggested that the left-brain cortex was more involved in the “retention” process of WM.

In contrast, a negative correlation was observed between RT and alpha frequency band, which indicated that the alpha oscillation ERSP values decreased when the RT differences between the rTMS and sham groups increased. This phenomenon was localized to the bilateral prefrontal area, left central area, left central-parietal area and left parietal area. The electrodes that had the significant negative correlation by rTMS treatment included F3 (*r* = −0.559, *P* < 0.01**), Fz (*r* = −0.726, *P* < 0.05*), F4 (*r* = −0.535, *P* < 0.01**), C3 (*r* = −0.527, *P* < 0.05*), CP1 (*r* = −0.607, *P* < 0.05*), CP3 (*r* = −0.52, *P* < 0.05*), CP5 (*r* = −0.661, *P* < 0.01**), P7 (*r* = −0.601, *P* < 0.05*), and P1 (*r* = −0.638, *P* < 0.01**). The most marked correlation was observed at Fz (Figure 6B), which was located at the prefrontal area. Similar brain regions exhibited significant correlations with the two frequency bands. In addition, the brain regions that correlated with the alpha frequency band covered a larger area than those that correlated with the theta frequency band.

### Phase Synchronization

Multiple brain areas, including the prefrontal area, central area, and parietal area are involved in the “retention” process of WM. However, it remains unclear whether connectivity exists between the parietal area and other brain areas following rTMS of the parietal lobe. Many prior studies have shown that rTMS could affect brain activities when the frequency in the brain region was similar to the stimulus frequency. In this study, 5-Hz rTMS enhanced theta-band amplitude and decreased alpha-band amplitude in different brain areas.

**Figure 7:**
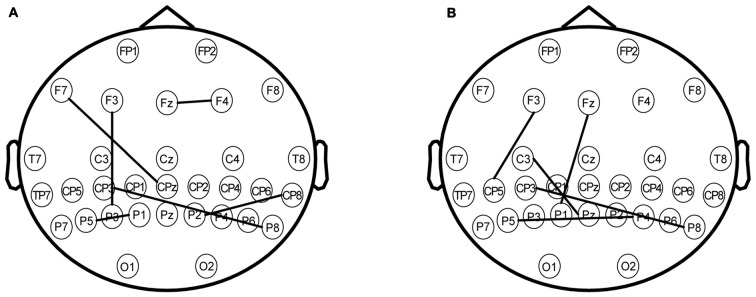
**(A)** The phase synchronization of electrodes under the theta frequency band. The significant difference between rTMS and sham with phase locking value (PLV) on the map (*P* < 0.01**) involved the following coupling brain areas: the prefrontal area (electrodes including F7, F3, Fz and F4), the parietal area (electrodes including CP3, CPz, TP8, P5, P3, P1, P2 and P8). **(B)** The phase synchronization of the electrodes under the alpha frequency band. The significant difference between rTMS and sham with PLV on the map (*P* < 0.01**) involved the following coupling brain areas: the left prefrontal area (electrodes including F3 and Fz), the left central area (electrodes including C3), the parietal area (electrodes including CP5, CP3, P5, P1, Pz, P4 and P8).

Via the PLV comparison between the two conditions (rTMS vs. sham), we found that phase synchronization between the parietal area and other brain areas became markedly stronger when the parietal lobe was stimulated by rTMS. For the theta oscillation, phase synchronization between the left parietal area and left prefrontal area was strengthened by rTMS (Figure 7A, *P* < 0.01**). A similar phase synchronism was found under alpha oscillation (Figure 7B, *P* < 0.01**). This finding suggested that the stimulation of the parietal brain region resulted in amplitude alterations of the prefrontal region during the “retention” period of WM.

## Discussion

In the present study, we first found that both the prefrontal and parietal areas were affected by rTMS treatment. The ERSP values of theta and alpha oscillations were changed by rTMS, while the variation was correlated with behavioral performance. Meanwhile, phase synchronism between the two brain areas was increased by rTMS. The connectivity between the two brain areas could reflect their cooperation.

### Oscillations Reflect Brain Activity

In EEG studies of WM, brain activities in the parietal and prefrontal areas were related to coding, maintenance and processing of information. Several studies have shown that theta oscillation ERSP value in the prefrontal area increases during learning and consolidation of associative memory (Jensen and Tesche, 2002; Meltzer et al., 2008; Takehara-Nishiuchi et al., 2012; Enriquez-Geppert et al., 2014). Other findings uncovered that theta oscillation ERSP values in the parietal-occipital and temporal areas increased during the entire WM process, which is called the “gate” effect (Raghavachari et al., 2006). In addition, other studies on theta oscillation have shown that changes in ERSP value reflect correlations between different cortical areas. For cognitive task, inter-site phase coherence of theta oscillation reflects the correlation between two cortical areas, such as the parietal and prefrontal areas (Sauseng et al., 2004; Sammer et al., 2007; Wu et al., 2007; Liebe et al., 2012). In this study, theta oscillation ERSP values in both the left parietal and left prefrontal areas were positively correlated with behavioral data (e.g., RT). Further analyses showed that rTMS enhanced the phase synchronizations between these two cortical regions, which may have strengthened the connections between these two regions.

In contrast, some studies have suggested that alpha oscillation ERSP values were reduced during WM (Meyer et al., 2013; Tenke et al., 2015). In our study, the rTMS, alpha oscillation ERSP values were negatively correlated with RT at different cortical regions, including the prefrontal area, right central area and parietal area, following rTMS. This result suggested that these cortical regions were activated during the “retention” process. Furthermore, alpha oscillation synchronization between the parietal area and other cortical regions (including the prefrontal and central areas) also reflected their co-operative relationship during the “retention” process following rTMS. Meanwhile, we observed that alpha oscillation was increased on the lateral parietal area to the stimulation location, but was not significant (Figure 5B). The result could be interpreted as inhibition of the interfering information in the task-irrelevant brain areas (Klimesch et al., 2003; Hamidi et al., 2009; Benedek et al., 2014).

### Was the Prefrontal Area Activated by rTMS?

In this study, the oscillation ERSP value data showed variations only at the parietal area, and not the prefrontal area, for both theta and alpha oscillations. However, while establishing the correlation between the ERSP value and RT differences, the prefrontal area was also found to be activated during the process. What is the mechanism by which the activity of the prefrontal area is induced? Is this activity connected to parietal area activity? Several recent studies have reported connections between these two cortices. Kundu et al. (2015) found increased directed connectivity between the prefrontal and parietal areas during the “retention” process by using the Granger causality of the alpha oscillation; however, similar results were also observed between both the prefrontal and occipital areas, and the parietal and occipital areas during the “coding” process. In addition, Harding et al. (2015) established a brain network model for cognitive control and WM, which included the inferior frontal junction, dorsolateral prefrontal cortex (DLPFC), pre-supplementary motor area, and intra-parietal sulcus, and analyzed the causality between different cortical regions via functional magnetic resonant imaging (fMRI).

Furthermore, several studies on rTMS have explained the effects of increased correlation between the parietal and prefrontal areas on WM task performance. Wang et al. (2014) designed an associative memory experiment to test cortical-hippocampal brain networks. The target location for stimulation was the SPL. The hippocampus is associated mainly with cognitive functions such as memory and judgment, which are also associated with the prefrontal cortex and parietal area. Using fMRI data, the findings of the study revealed an association between hippocampal activations and better task performances, which suggested that cortical regions connected with the parietal area might be indirectly activated by parietal area stimulations. In our study, the PLV analysis results (Figures 7A,B) also showed there was synchronization between the parietal and prefrontal areas that was strengthened by rTMS. Moreover, the brain areas that showed synchronization were similar to those that showed correlations between oscillation ERSP value and behavioral data. These results indicated that the EEG activities in the prefrontal area were strongly correlated to the EEG activities in the parietal area during the WM task. Using rTMS, the prefrontal area may be activated indirectly by stimulating the parietal lobe. We had hypothesized that the rTMS may induce changes in EEG oscillations in regions of brain, and that the EEG phase synchronizations between these cortices would be enhanced by rTMS.

### The Correlation between Behavior and EEG Activity

Previous studies have compared behavior performance and EEG activities of WM task participants between the rTMS and control groups. However, the original EEG data could not directly reflect whether the brain’s response was positive or negative. In our study, the positive behavioral performances (e.g., decrease in RT) were correlated with the EEG activities (theta or alpha band oscillations), which made the EEG analysis more functional. By investigating the correlation between behavior and EEG activity of different brain areas, we could find which cortical brain regions were involved in the responses of WM tasks.

In the present work, subjects achieved better performances of WM tasks with 5-Hz rTMS of the parietal area, while their theta band oscillation increased more in the left parietal and left prefrontal brain regions, and the alpha band oscillation decreased more in the left parietal and bilateral prefrontal brain regions. According to the EEG oscillation activities of the subjects, the left-brain region, including the left parietal and prefrontal areas, was affected by the action of 5-Hz rTMS. Therefore, these regions were more involved in brain activity during the “retention” period of WM, which surely increased their task performances.

### Limitation

In the spectral analysis, the preprocessing method contains two baseline removal procedures, which are referenced from Hamidi et al. (2008), and the method is also used in Makeig (1993) and other documents. In the first baseline removal procedure, the ERSP data during the “retention” period were obtained by ERSP analysis. In the second baseline removal procedure, the normalized ERSP data were obtained by subtracting the interval-period ERSP data from the ERSP data during the “retention” period. The main idea of this method through two processes, respectively, to remove short-term and long-term tasks irrelevant components. The traditional ERSP calculation can only remove task before and after the execution of a task independent component, but as everyone knows, brain activity will be changed greatly after a relatively long interval. Since the experiment took more than 1 h, the process which took a long time may be brought serious impact for the brain activity. In this study, the baseline removal procedure was divided the two steps, respectively, to remove irrelevant components of the task. However, this process may cause some useful components to be removed. This requires further research to evaluate and improve.

The calculation of PLV was affected by several factors. Because of the existence of the volume conductor effect, signals collected on either electrode could be considered as a synthesis of the electrical activity of all neurons. Therefore, the signals of any two electrodes had a lot of common components, which made the PLV higher than the actual value. The solution was to map the multichannel EEG back to the cerebral cortex by means of traceability analysis. At the level of the cortex, the coherence of electrical activity between the two brain regions was examined and this connection between the two brain regions could be described more accurately than that in the electrode space. However, there are some problems with this method, for example, most of the trace back algorithms take many simplifications, and the obtained electrical activity information was a statistical estimation result. In addition, different algorithms achieved inconsistent results, which add to the difficulty of the analysis. It is also important that, due to the existence of the volume conductor effect, the EEG signals actually acquired by the epidermal electrodes were convolution of many sources rather than simple linear sum. The estimation of the traceability algorithm was rarely taken into account at present, so the accuracy of traceability was still to be improved. The computation of PLV also had a problem of time window, in which the EEG is assumed to be stationary, and this will lead to some estimation errors. In conclusion, both the time-frequency features of ERSP and the phase synchronization of PLV were affected by many factors, and these results had some limitations. More comprehensive analyses should be made with reference to studies of multiple neurophysiological signals, such as functional magnetic resonance imaging (fMRI) or functional near-infrared spectroscopy (fNIRS).

## Conclusion

In this study, we analyzed variations in different brain areas of participants during the “retention” process of WM tasks following 5-Hz rTMS. The conclusions were as follows:

1. Both the prefrontal and parietal areas were affected by rTMS. The EEG theta band (4–7 Hz) oscillation was increased and the alpha band (8–14 Hz) oscillation was decreased by the rTMS. The ERSP value variation of brain oscillations have correlated with the RT variation. By using rTMS, the more RT decreased, the more ERSP value of theta oscillation increased. On the contrary, the more RT decreased, the more ERSP value of alpha oscillation decreased.
2. The brain activities in left parietal and prefrontal areas seem to exhibited greater synchronization in the theta and alpha band oscillations, suggesting that the connectivity between the two areas became stronger after rTMS.

We may conjecture that, given the connection between the parietal and prefrontal areas, rTMS may indirectly activate the prefrontal area through stimulation of the parietal area. The activities in these two cortical regions could then have cooperated to maintain information. Additionally, the activity synchronization between these two regions may enhance our understanding of the mechanisms underlying the effects of rTMS. Our study provides more information regarding brain activity during the “retention” process of WM, and will facilitate further studies into human cognitive processes.

## Author Contributions

SL, J-NJ and XW were responsible for the design of the work, and completed the experiment for acquiring the EEG data. SL, J-NJ, H-ZQ and Z-PL completed the analysis and the interpretation of the data. All of the authors participated in drafting the work. SL, Z-PL and TY were responsible for revising the important intellectual content involved in the article and approved the final version of the article.

## Conflict of Interest Statement

The authors declare that the research was conducted in the absence of any commercial or financial relationships that could be construed as a potential conflict of interest.

## References

[B1] Baddeley A. D. Chincotta D. Adlam A. (2001). Working memory and the control of action: evidence from task switching. J. Exp. Psychol. Gen. 130, 641–657. 10.1037/0096-3445.130.4.641 11757873

[B2] Benedek M. Schickel R. J. Jauk E. Fink A. Neubauer A. C. (2014). Alpha power increases in right parietal cortex reflects focused Internal attention. Neuropsychologia 56, 393–400. 10.1016/j.neuropsychologia.2014.02.010 24561034 PMC3989020

[B4] Delorme A. Makeig S. (2004). EEGLAB: an open source toolbox for analysis of single-trial EEG dynamics including independent component. J. Neurosci. Methods 134, 9–21. 10.1016/j.jneumeth.2003.10.009 15102499

[B5] Eggers J. Bauml R. Tzschoppe R. Girod B. (2003). Scalar costa scheme for information embedding. IEEE Trans. Signal Proces. 51, 1003–1019. 10.1109/tsp.2003.809366

[B48] Enriquez-Geppert S. Huste R. J. Figge C. Herrmann C. S. (2014). Self-regulation of frontal-midline theta facilitates memory updating and mental set shifting. Front. Behav. Neurosci. 8:420. 10.3389/fnbeh.2014.00420 25538585 PMC4257088

[B7] Fisher R. A. (1935). The Design of Experiment. New York, NY: Hafner Press.

[B8] Gevins A. Smith M. E. McEvoy L. Yu D. (1997). High-resolution EEG mapping of cortical activation related to working memory: effects of task difficulty, type of processing, and practice. Cereb. Cortex 7, 374–385. 10.1093/cercor/7.4.374 9177767

[B9] Hamidi M. Feredoes E. Tononi G. Postle B. R. (2008). Assessment of long-term, within-session effects of high-frequency repetitive transcranial magnetic stimulation on a cognitive task. Brain Stimul. 3, 266–267. 10.1016/j.brs.2008.06.012

[B10] Hamidi M. Slagter H. A. Tononi G. Postle B. R. (2009). Repetitive transcranial magnetic stimulation affects behavior by biasing endogenous cortical oscillations. Front. Integr. Neurosci. 3:14. 10.3389/neuro.07.014.2009 19587850 PMC2707056

[B11] Hamidi M. Slagter H. A. Tononi G. Postle B. R. (2010). Brain responses evoked by high-frequency repetitive transcranial magnetic stimulation: an event-related potential study. Brain Stimul. 3, 2–14. 10.1016/j.brs.2009.04.001 20383278 PMC2850212

[B12] Harding I. H. Yücel M. Harrison B. J. Pantelis C. Breakspear M. (2015). Effective connectivity within the frontoparietal control network differentiates cognitive control and working memory. Neuroimage 106, 144–153. 10.1016/j.neuroimage.2014.11.039 25463464

[B13] Hsieh L.-T. Ekstrom A. D. Ranganath C. (2011). Neural oscillations associated with item and temporal order maintenance in working memory. J. Neurosci. 31, 10803–10810. 10.1523/jneurosci.0828-11.2011 21795532 PMC3164584

[B14] Hsieh L.-T. Ranganath C. (2014). Frontal midline theta oscillations during working memory maintenance and episodic encoding and retrieval. Neuroimage 85, 721–729. 10.1016/j.neuroimage.2013.08.003 23933041 PMC3859771

[B15] Jensen O. Mazaheri A. (2010). Shaping functional architecture by oscillatory alpha activity: gating by inhibition. Front. Hum. Neurosci. 4:186. 10.3389/fnhum.2010.00186 21119777 PMC2990626

[B17] Jensen O. Tesche C. D. (2002). Frontal theta activity in humans increases with memory load in a working memory task. Eur. J. Neurosci. 15, 1395–1399. 10.1046/j.1460-9568.2002.01975.x 11994134

[B18] Kang J.-S. Ukeob P. Gonuguntla V. Veluvolu K. C. Minho L. (2015). Human implicit intent recognition based on the phase synchrony of EEG signals. Pattern Recognit. Lett. 66, 144–152. 10.1016/j.patrec.2015.06.013

[B20] Kardos Z. Tóth B. Boha R. File B. Molnár M. (2014). Age-related changes of frontal-midline theta is predictive of efficient memory maintenance. Neuroscience 273, 152–162. 10.1016/j.neuroscience.2014.04.071 24846615

[B21] Klimesch W. Sauseng P. Gerloff C. (2003). Enhancing cognitive performance with repetitive transcranial magnetic stimulation at human individual alpha frequency. Eur. J. Neurosci. 17, 1129–1133. 10.1046/j.1460-9568.2003.02517.x 12653991

[B22] Klimesch W. Sauseng P. Hanslmayr S. (2007). EEG alpha oscillations: the inhibition-timing hypothesis. Brain Res. Rev. 53, 63–88. 10.1016/j.brainresrev.2006.06.003 16887192

[B23] Krause C. M. Sillanmäki L. Koivisto M. Saarela C. Häggqvist A. Laine M.. (2000). The effects of memory load on event-related EEG desynchronization and synchronization. Clin. Neurophysiol. 111, 2071–2078. 10.1016/s1388-2457(00)00429-6 11068244

[B24] Kundu B. Chang J.-Y. Postle B. R. Van Veen B. D. (2015). Context-specific differences in fronto-parieto-occipital effective connectivity during short-term memory maintenance. Neuroimage 114, 320–327. 10.1016/j.neuroimage.2015.04.001 25863155 PMC4446161

[B25] Lachaux J.-P. Rodriguez E. Martinerie J. Varela F. J. (1999). Measuring phase synchrony in brain signals. Hum. Brain Mapp. 8, 194–208. 10.1002/(SICI)1097-0193(1999)8:4<194::AID-HBM4>3.0.CO;2-C 10619414 PMC6873296

[B26] Liebe S. Hoerzer G. M. Logothetis N. K. Rainer G. (2012). Theta coupling between V4 and prefrontal cortex predicts visual short-term memory performance. Nat. Neurosci. 15, 456–462. 10.1038/nn.3038 22286175

[B27] Luber B. Kinnunen L. H. Rakitin B. C. Ellsasser R. Stern Y. Lisanby S. H. (2007). Facilitation of performance in a working memory task with rTMS stimulation of the precuneus: frequency- and time-dependent effects. Brain Res. 1128, 120–129. 10.1016/j.brainres.2006.10.011 17113573

[B28] Luber B. Lisanby S. H. (2014). Enhancement of human cognitive performance using transcranial magnetic stimulation (TMS). Neuroimage 85, 961–970. 10.1016/j.neuroimage.2013.06.007 23770409 PMC4083569

[B29] Makeig S. (1993). Auditory event-related dynamics of the EEG spectrum and effects of exposure to tones. Electroencephalogr. Clin. Neurophysiol. 86, 283–293. 10.1016/0013-4694(93)90110-h 7682932

[B31] Meltzer J. A. Zaveri H. P. Goncharova I. I. Distasio M. M. Papademetris X. Spencer S. S.. (2008). Effects of working memory load on oscillatory power in human intracranial EEG. Cereb. Cortex 18, 1843–1855. 10.1093/cercor/bhm213 18056698 PMC2474453

[B32] Meyer L. Obleser J. Friederici A. D. (2013). Left parietal alpha enhancement during working memory-intensive sentence processing. Cortex 49, 711–721. 10.1016/j.cortex.2012.03.006 22513340

[B33] Michels L. Moazami-Goudarzi M. Jeanmonod D. Sarnthein J. (2008). EEG alpha distinguishes between cuneal and precuneal activation in working memory. Neuroimage 40, 1296–1310. 10.1016/j.neuroimage.2007.12.048 18272404

[B37] Pesarin F. (1992). A resampling procedure for nonparametric combination of several depend test. J. Ital. Stat. Soc. 1, 87–101. 10.1007/bf02589052

[B38] Pitman E. J. G. (1938). Significance tests which may be applied to samples from any populations. Part III. The analysis of variance test. Biometrika 29, 322–335. 10.2307/2332008

[B39] Postle B. R. Ferrarelli F. Hamidi M. Feredoes E. Massimini M. Peterson M.. (2006). Repetitive transcranial magnetic stimulation dissociates working memory manipulation from retention functions in prefrontal, but not posterior parietal, cortex. J. Cogn. Neurosci. 18, 1712–1722. 10.1162/jocn.2006.18.10.1712 17014375

[B40] Raghavachari S. Kahana M. J. Rizzuto D. S. Caplan J. B. Kirschen M. P. Bourgeois B.. (2001). Gating of human theta oscillations by a working memory task. J. Neurosci. 21, 3175–3183. 11312302 10.1523/JNEUROSCI.21-09-03175.2001 PMC6762557

[B41] Raghavachari S. Lisman J. E. Tully M. Madsen J. R. Bromfield E. B. Kahana M. J. (2006). Theta oscillations in human cortex during a working-memory task: evidence for local generators. J. Neurophysiol. 95, 1630–1638. 10.1152/jn.00409.2005 16207788

[B42] Rogasch N. C. Thomson R. H. Farzan F. Fitzgibbon B. M. Bailey N. W. Hernandez-Pavon J. C.. (2014). Removing artefacts from TMS-EEG recordings using independent component analysis: importance for assessing prefrontal and motor cortex network properties. Neuroimage 101, 425–439. 10.1016/j.neuroimage.2014.07.037 25067813

[B43] Sammer G. Blecker C. Gebhardt H. Bischoff M. Stark R. Morgen K.. (2007). Relationship between regional hemodynamic activity and simultaneously recorded EEG-Theta associated with mental arithmetic-induced workload. Hum. Brain Mapp. 28, 793–803. 10.1002/hbm.20309 17080437 PMC6871320

[B44] Sauseng P. Griesmayr B. Freunberger R. Klimesch W. (2010). Control mechanisms in working memory: a possible function of EEG theta oscillations. Neurosci. Biobehav. Rev. 34, 1015–1022. 10.1016/j.neubiorev.2009.12.006 20006645

[B45] Sauseng P. Klimesch W. (2008). What does phase information of oscillatory brain activity tell us about cognitive processes? Neurosci. Biobehav. Rev. 32, 1001–1013. 10.1016/j.neubiorev.2008.03.014 18499256

[B46] Sauseng P. Klimesch W. Doppelmayr M. Hanslmayr S. Schabus M. Gruber W. R. (2004). Theta coupling in the human electroencephalogram during a working memory task. Neurosci. Lett. 354, 123–126. 10.1016/j.neulet.2003.10.002 14698454

[B47] Scheeringa R. Petersson K. M. Oostenveld R. Norris D. G. Hagoort P. Bastiaansen M. C. M. (2009). Trial-by-trial coupling between EEG and BOLD identifies networks related to alpha and theta EEG power increases during working memory maintenance. Neuroimage 44, 1224–1238. 10.1016/j.neuroimage.2008.08.041 18840533

[B49] Stockes M. G. Chambers C. D. Gould I. C. Henderson T. R. Janko N. E. Allen N. B.. (2006). Simple metric for scaling motor threshold based on scalp-cortex distance: application to studies using transcranial magnetic stimulation. J. Neurophysiol. 94, 4520–4527. 10.1152/jn.00067.2005 16135552

[B19] Takehara-Nishiuchi K. Maal-Bared G. Morrissey M. D. (2012). Increased entorhinal-prefrontal theta synchronization parallels decreased entorhinal-hippocampal theta synchronization during learning and consolidation of associative memory. Front. Behav. Neurosci. 5:90. 10.3389/fnbeh.2011.00090 22319482 PMC3262397

[B50] Tenke C. E. Kayser J. Abraham K. Alvarenga J. E. Bruder G. E. (2015). Posterior EEG alpha at rest and during task performance: comparison of current source density and field potential measures. Int. J. Psychophysiol. 97, 299–309. 10.1016/j.ijpsycho.2015.05.011 26026372 PMC4537800

[B51] Tesche C. D. Karhu J. (2000). Theta oscillations index human hippocampal activation during a working memory task. Proc. Natl. Acad. Sci. U S A 97, 919–924. 10.1073/pnas.97.2.919 10639180 PMC15431

[B52] Tokariev A. Vanhatalo S. Palva J. M. (2016). Analysis of infant cortical synchrony is constrained by the number of recording electrodes and the recording montage. Clin. Neurophysiol. 127, 310–323. 10.1016/j.clinph.2015.04.291 26122070

[B34] van Mierlo P. V. Papadopoulou M. Carrette E. Boon P. Vandenberghe S. Vonck K.. (2014). Functional brain connectivity from EEG in epilepsy: seizure prediction and epileptogenic focus localization. Prog. Neurobiol. 121, 19–35. 10.1016/j.pneurobio.2014.06.004 25014528

[B53] Veniero D. Bortoletto M. Miniussi C. (2009). TMS-EEG co-registration: on TMS-induced artifact. Clin. Neurophysiol. 120, 1392–1399. 10.1016/j.clinph.2009.04.023 19535291

[B55] Wang J. X. Rogers L. M. Gross E. Z. Ryals A. J. Dokucu M. E. Brandstatt K. L.. (2014). Targeted enhancement of cortical-hippocampal brain networks and associative memory. Science 345, 1054–1057. 10.1126/science.1252900 25170153 PMC4307924

[B56] Wu X. Chen X. Li Z. Han S. Zhang D. (2007). Binding of verbal and spatial information in human working memory involves large-scale neural synchronization at theta frequency. Neuroimage 35, 1654–1662. 10.1016/j.neuroimage.2007.02.011 17379539

[B57] Yamanaka K. Yamagata B. Tomioka H. Kawasaki S. Mimura M. (2009). Transcranial magnetic stimulation of the parietal cortex facilitates spatial working memory: near-infrared spectroscopy study. Cereb. Cortex 20, 1037–1045. 10.1093/cercor/bhp163 19684247

